# Molecular detection and genetic characterization of *Trichomonas gallinae* in falcons in Saudi Arabia

**DOI:** 10.1371/journal.pone.0241411

**Published:** 2020-10-29

**Authors:** Abdulwahed Fahad Alrefaei

**Affiliations:** Department of Zoology, King Saud University, College of Science, Riyadh, Saudi Arabia; Temple University, UNITED STATES

## Abstract

Avian trichomonosis is primarily caused by *Trichomonas gallinae*, a flagellated protozoan parasite that especially infects the upper digestive tract of columbid bird species and their avian predators. However, this parasite has recently been found to be distributed worldwide in various other avian species. This parasitic disease is common in captive falcons in Saudi Arabia and the Middle East. This study aimed to examine and identify the genetic variation of *T*. *gallinae* obtained from three species of falcons in Saudi Arabia via the sequencing analysis of the internal transcribed spacer (ITS) region. Swab samples from 97 saker falcons (*Falco cherrug*), 24 peregrine falcons (*Falco peregrinus*) and 37 gyrfalcons (*Falco rusticolus*) were cultured and analysed for infection between 2018 and 2019. The overall prevalence of infection by *T*. *gallinae* was 26.58% (n = 42), of which 35 (83.33%) were collected from Riyadh region and seven (16.67%) were collected from Qassim region. The results indicate the presence of four genotypes of *T*. *gallinae* in Saudi falcons: A, C, II, and KSA11. This study reports for the first time genetic diversity of *T*. *gallinae* in these falcons in Saudi Arabia.

## Introduction

Avian trichomonosis is a parasitic disease caused by the flagellated protozoan parasite *Trichomonas gallinae* [1]. This parasite infects the upper digestive tract of domestic and wild birds, including the mouth, crop, oesophagus and pharynx, causing erosions and necrotic lesions, as well as necrotic foci within internal organs, such as the liver and lungs. Severe cases can be deadly in the early days following infection [2,3]. Columbid species are considered the principal reservoir hosts of *T*. *gallinae* [1]. In addition to Columbiformes, several other bird species worldwide are infected by this parasite, including raptors, gallinaceous birds and passerines [4–8].

The main route of transmission of *T*. *gallinae* between birds includes the feeding of squabs by an adult, normal feeding via courtship billing during nesting, sharing contaminated food or water sources, and via predation and necrophagy from raptors in the wild [9,10]. *Trichomonas gallinae* in captive falcons in Saudi Arabia and Middle East seem to be related to the traditional Arabian falconry practices of training falcons using live or freshly domestic pigeons [11,12].

Recently, *T*. *gallinae* was reported as an etiologic agent of epidemic outbreaks of disease affecting wild birds across Europe and Canada [2,13,14]. This disease can cause significant effects at the population level, especially for naïve populations. It is considered a major threat to the endangered pink pigeon (*Columba mayeri*) in Mauritius [15].

There are several studies from Europe and North America on the prevalence, virulence and genetic diversity of avian trichomonads, but reports from Asia and the Middle East are rare [16,17]. The genetic diversity of *T*. *gallinae* and its correlation with clinical presentations correlated to the sequencing of the internal transcribed spacer (ITS) region performed in Europe, the UK and the USA have revealed a relationship between the genetic variation and the presence of necrotic ingluvitis lesions [5,13,18–21]. In a recent study, sequencing was performed on 15 distinct ITS genotypes within *T*. *gallinae* isolates from pigeons, common mynas *(Acridotheres tristis)*, chickens and turkeys in Riyadh, Saudi Arabia [8].

This study aimed to examine the genetic diversity of *T*. *gallinae* obtained from different falcon species in Saudi Arabia via the sequence analysis of 5.8S ribosomal RNA (rRNA) and its flanking ITS regions 1 and 2 (ITS1 and ITS2).

## Materials and methods

### Sources of isolates

Oral swab samples from 158 falcons were tested in the context of the routine diagnosis of captive falcons in the Al-Qassim Falcon Center in Unaizah (26°07'39.4"N 43°57'20.3"E), the Falcon Medical and Research Hospital of the Fahad bin Sultan Falcon Center in Riyadh (24°46'19.3"N 46°43'09.8"E) and during the first Saudi Falcons and Hunting Exhibition in Riyadh (24°48'52.4"N 46°37'15.4"E) from December 2018 and through February 2020 (Table 1). “All procedures for the samples collection were carried out in strict accordance with the recommendations by the Research Ethics Sub-Committee (REC) of the College of Sciences at the King Saud University (KSU) in Riyadh, Kingdom of Saudi Arabia (KSA) (Ethics Reference No: KSU-SE-19-77)”. The ethics committee has approved the current study. The sample collection procedures were conducted under direct veterinary supervision. Clinical signs and descriptions of any lesions were recorded for each examined bird. The falcons collected from the centers were anesthetised with isoflurane via a facemask before undergoing an endoscopic examination of the upper digestive tract to look for the presence of macroscopic lesions. The falcons collected from the Saudi Falcons and Hunting Exhibition were examined for the presence of oropharyngeal trichomonosis lesions by direct inspection. Veterinarians used sterile cotton swabs to gently swab live falcons over approximately 120° to match the natural anatomical curvature of the oral cavity, oropharynx and crop. All samples were subsequently cultured at 37°C in 5 ml of fresh trypticase-yeast extract-maltose (TYM) medium containing 10% inactivated horse serum (Sigma-Aldrich, St. Louis, Missouri, USA), 100X antibiotic-antimycotic (10,000 units/mL penicillin + 10,000 μg/mL streptomycin + 25 μg/mL amphotericin B; UFC Biotech, KSA) at pH 7.2. All samples were examined under a microscope at 10X magnification at 24 hours post-incubation and thereafter at 24-hour intervals for up to five days to monitor for the motility of *T*. *gallinae*. The infection status of the samples was determined to be positive if motile *T*. *gallinae* were found in any of the culture tests for an individual bird, and the samples were designated as negative if no parasites were detected after five days of incubation. The positive samples were stored with 5% DMSO (Sigma-Aldrich) at −80°C for any additional work.

**Table 1:** *Trichomonas gallinae* prevalence in falcons.

Region	Species	No. tested	No. positive	Prevalence (%)
Riyadh	Saker falcon *F*. *cherrug*	75^A^	14	18.66
	Gyrfalcon *F*. *rusticolus*	27^A^	13	48.14
	Peregrine falcon *F*. *peregrinus*	13	8	61.54
Qassim	Saker falcon *F*. *cherrug*	22	2	9
	Gyrfalcon *F*. *rusticolus*	10	3	30
	Peregrine falcon *F*. *peregrinus*	11	2	18.18

^A^ 31 Saker and 4 Gyrfalcon were collected from Saudi Falcons and Hunting Exhibition in Riyadh.

### DNA extraction

DNA was extracted from the positive culture isolates using DNAzol® reagent (Invitrogen, UK) under the following conditions. The TYM medium containing *T*. *gallinae* was transferred to a 1-ml Eppendorf tube and centrifuged at 12,000 rpm for 5 min; the supernatant was removed and the pellet retained; 500 μl of DNAzol® was added to each sample and briefly pipetted to rapidly lyse the cells and the samples were centrifuged at 10,000 rpm for 2 min at 4°C. The supernatant was transferred to a fresh Eppendorf tube, the pellets discarded, and 500 μl of 100% ethanol added to the supernatant and vortexed, which was then centrifuged for 5 min at 10,000 rpm at 4°C to precipitate the DNA. The supernatant was discarded, and the DNA pellet was washed in 70% ethanol and centrifuged at 10,000 rpm for 3 min. The new supernatant was removed and the DNA pellet air-dried for 10 min before adding 100 μL of nuclease-free water to dissolve the DNA. The extracted DNA was stored at −20°C for future work if necessary. The DNA concentrations for each isolate were measured using spectrophotometry with an absorbance of 260 nm. The extracted DNA was electrophoresed through a 1% agarose gel stained with ethidium bromide and visualised using UV transillumination.

### PCR amplification of the ITS1/5.8S/ITS2 fragment

The PRC analysis was carried out using forward primer TFR1 (5′-TGCTTCAGTTCAGCGGGTCTTCC-3′) and reverse primer TFR2 (5′-CGGTAGGTGAACCTGCCGTTGG-3′) [22,23]. The reaction was conducted in a 25-μL total volume comprising 8.5 μL Green Master Mix (2X, USA), 3 μL each of forward and reverse primers, 8.5 μL ddH_2_O and 2 μL of genomic DNA. The cycling parameters consisted of an initial denaturation at 98°C for 5 min, 45 cycles at 98°C for 30 s, annealing at 61°C for 30 s with an extension at 72°C for 1 min, and a final extension step at 72°C for 5 min. The PCR products were electrophoresed in 1% agarose gel.

### Sequence analysis and phylogenetic trees

The evolutionary relationships among the sequences of *T*. *gallinae* were compared by building a phylogenetic tree using MEGA, Version 7, [24] and CLUSTALX, Version 2.1, software [25]. All sequence data for the ITS regions were inspected and refined using the MEGA trace data file viewer/editor, and then aligned using the forward and reverse complement of the reverse primer. All sequences obtained in this study were uploaded in GenBank under the accession number MT300158-MT300161 and MW114451-MW114485. A number of reference sequences were downloaded from the National Center for Biotechnology Information (NCBI) GenBank database for comparison with the ITS regions. The phylogenetic trees of the datasets obtained from GenBank and those identified in this study were constructed separately using the neighbour-joining method with genetic distance and Tamura-Nei models. These were used to analyse the relationships between taxa via nucleotide sequences [24,25]. Felsenstein’s bootstrap method was used to calculate the associated taxa clustered in the bootstrap test (1,000 replicates).

## Results

In total, 158 oral swab samples were collected from three different falcon species in Riyadh and Qassim from December 2018 through February 2020. Forty-two (26.58%) samples tested microscopically positive for *T*. *gallinae*. Of the positive samples, 35 (83.33%) and seven (16.67%) were collected from Riyadh and Qassim, respectively. The prevalence of falcons infected by *T*. *gallinae* is summarised by region and species in Table 1.

**Fig 1:**
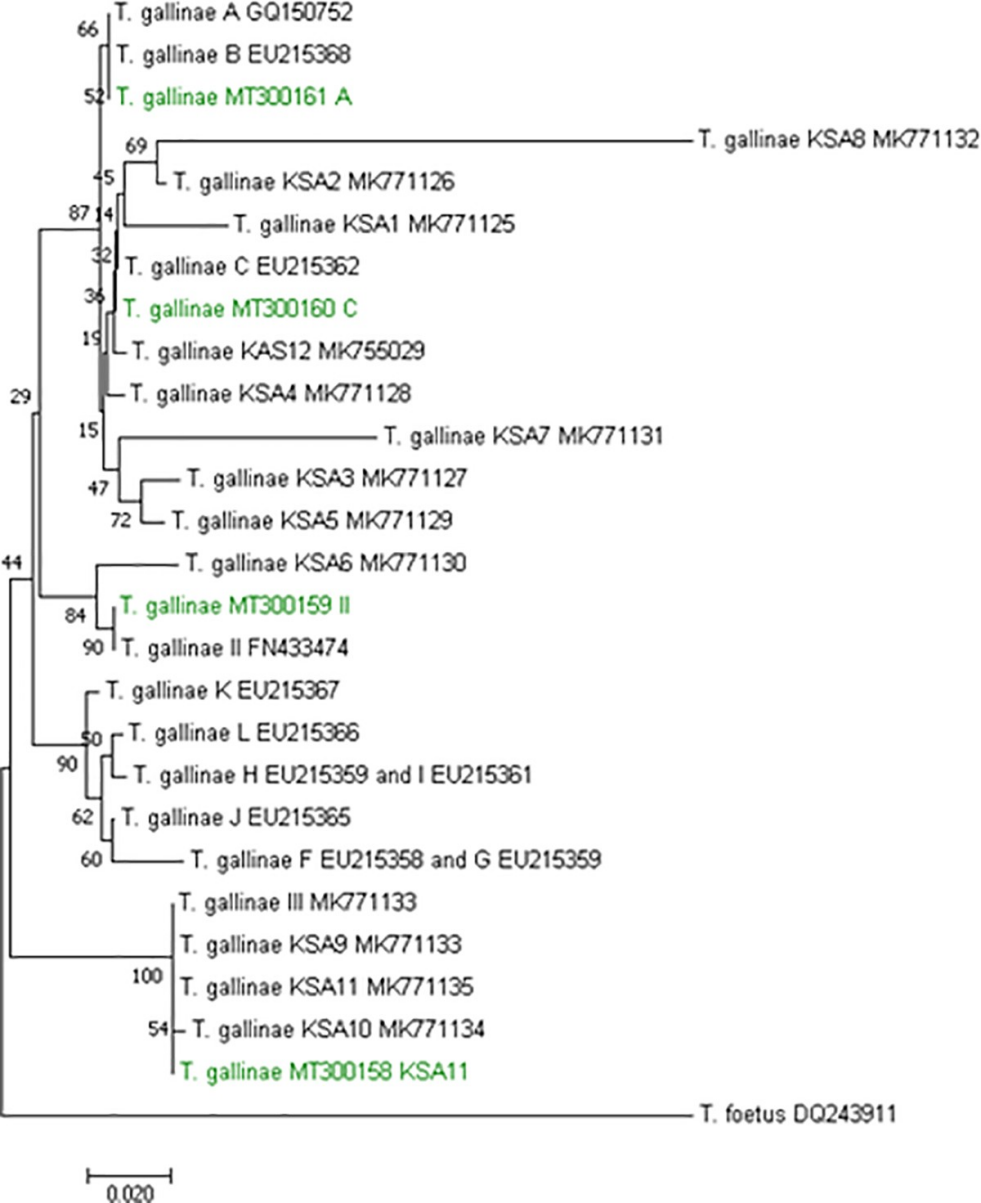
Phylogenetic tree based on ITS region indicating the relationship of *Trichomonas gallinae* genotypes using the NJ method. References to GenBank accession numbers are as follows: GQ150752 [13]; EU215368, EU215362, EU215358, EU215360, EU215365, EU215367, EU215366 [18]; FN433474 and FN433473 [26]; MK771125, MK771126, MK771127, MK771128, MK771129, MK771130, MK771131, MK771132, MK771133, MK771134, MK771135, and MK765029 [8]. *Tritrichomonas foetus* GenBank accession number DQ243911 [27] was included in the present study as an outgroup. The sequences identified in this study are shown in green.

The ITS1/5.8S/ITS2 region was successfully amplified in 39 samples using PCR, obtaining a fragment within this region of about 252 bp (Table 2). The genetic relationships among the *T*. *gallinae* were determined by the phylogenetic analysis of this ITS region. Four genotypes were found in this study among the 39 isolate sequences: A (n = 14), 157 C (n = 8), II (n = 14) and KSA11 (n = 3) (Fig 1). All of these *T*. *gallinae* genogroups have been previously described. Fourteen isolates were clustered in lineage II, which was generated from the sequence of an isolate obtained from a racing pigeon (GenBank: FN433474) in Austria [26]. This included the saker falcons (*Falco cherrug*) (n = 6), peregrine falcons (*F*. *peregrinus*) (n = 2) and gyrfalcons (*F*. *rusticolus*) (n = 6). Fourteen samples, obtained from *F*. *cherrug* (n = 8), *F*. *peregrinus* (n = 3) and *F*. *rusticolus* (n = 3), showed 100% identity to *T*. *gallinae* sequence A, previously reported in pigeons and finches (GenBank: GQ150752) in the UK [13] and birds of prey (GenBank: EU881912) in Spain [19]. In this study, eight samples, including *F*. *cherrug* (n = 2), *F*. *peregrinus* (n = 1) and *F*. *rusticolus* (n = 5), shared 100% identity to *T*. *gallinae* sequence C, previously reported in pigeons (GenBank EU215362) in the USA [18]. Only three *T*. *gallinae* isolates, obtained from *F*. *cherrug* (n = 1) and *F*. *peregrinus* (n = 2), showed 100% identity to the KSA11 sequence originally obtained from a feral pigeon in Saudi Arabia (GenBank MK771135) [8].

**Table 2:** Sample details of captive falcons. List of species, geographical location, evidence of lesions, and ITS sequence of *Trichomonas gallinae*. All samples were obtained from adult birds, collected from Qassim and Riyadh region in Saudi Arabia.

Isolation ID	Case no.	Host species	Location	Lesions	ITS sequence
1	Q9	Saker falcon	Qassim	+^A^	A
2	Q10	Gyrfalcon	Qassim	-	C
3	Q25	Peregrine falcon	Qassim	-	KSA11
4	Q26	Peregrine falcon	Qassim	-	II
5	Q28	Saker falcon	Qassim	-	A
6	Q35	Gyrfalcon	Qassim	-	II
7	Q38	Saker falcon	Qassim	-	II
8	R1	Saker falcon	Riyadh	-	C
9	R2	Peregrine falcon	Riyadh	+^A^	A
10	R3	Peregrine falcon	Riyadh	-	KSA11
11	R4	Gyrfalcon	Riyadh	-	II
12	R5	Gyrfalcon	Riyadh	-	C
13	R6	Saker falcon	Riyadh	-	A
14	R7	Gyrfalcon	Riyadh	-	II
15	R8	Gyrfalcon	Riyadh	+^A^	A
16	R9	Saker falcon	Riyadh	-	II
17	R10	Gyrfalcon	Riyadh	-	II
18	R12	Peregrine falcon	Riyadh	-	A
19	R15	Saker falcon	Riyadh	-	II
20	R16	Gyrfalcon	Riyadh	-	A
21	R17	Gyrfalcon	Riyadh	-	C
22	R18	Saker falcon	Riyadh	-	A
23	R19	Peregrine falcon	Riyadh	-	II
24	R20	Gyrfalcon	Riyadh	+^A^	II
25	R21	Peregrine falcon	Riyadh	-	C
26	R22	Saker falcon	Riyadh	+^A^	C
27	R23	Saker falcon	Riyadh	+^A^	II
28	R24	Saker falcon	Riyadh	-	A
29	R25	Peregrine falcon	Riyadh	-	A
30	R26	Gyrfalcon	Riyadh	-	C
31	R27	Gyrfalcon	Riyadh	-	C
32	R28	Saker falcon	Riyadh	-	A
33	R29	Saker falcon	Riyadh	-	II
34	R30	Saker falcon	Riyadh	-	KSA11
35	R31	Gyrfalcon	Riyadh	-	II
36	R32	Saker falcon	Riyadh	-	II
37	R33	Saker falcon	Riyadh	-	A
38	R34	Saker falcon	Riyadh	-	A
39	R35	Gyrfalcon	Riyadh	-	A

^A^Oral cavity lesions.

## Discussion

*Trichomonas gallinae* infects captive and wild bird species worldwide and is the causative agent of avian trichomonosis. This disease is important because it affects a number of different bird species, is characterised by great variation between different *T*. *gallinae* isolates, and may have conservation implications for at-risk falcon species, particularly those facing multiple threats. In this study, the ITS1/5.8S rRNA/ITS2 region was used to analyse genetic variation of *T*. *gallinae* isolated from three different species of falcon in Saudi Arabia. To the best of my knowledge, this is the first study at the genetic level to characterizing the genotypes of *T*. *gallinae* isolated from different falcon species in Saudi Arabia.

The four types of sequences detected in the current study correspond to four genotypes previously reported (Fig 1). These sequences, A, C, II and KSA11, are distinct lineages of *T*. *gallinae*, and all the sequences are commonly found in many species of birds, including columbids, raptors, chickens and finches, in various European countries, such as the UK, the USA, Canada, Brazil and Saudi Arabia [5,6,8,13,18,19,23,28,29]. However, the most important finding is that sequence II, originally reported in a racing pigeon in Austria (GenBank: FN433474) [26], was the most prevalent in the Saudi falcons. The results indicate that within the group of bird with lesions (6 of 39; 15%), genotype A is the most frequent (3 of 6; 50%). This data is consistent with the results reported by Robinson et al. (2010) [9], who found genotype A most often in birds with pathognomonic lesions.

These data support a wide range of hosts for *T*. *gallinae*, including birds of prey that feed on columbids, such as the feral pigeon *(Columba livia)*. Albeshr and Alrefaei (2020) [8] reported that sequence analyses of *T*. *gallinae* obtained from different species of birds in Riyadh showed at least 15 unique sequences, which were clearly divided into different branches depending on the ITS region sequence. However, all the sequences found in the current study (i.e. A, C, II and KSA11) were previously found only in pigeons within Saudi Arabia.

These results are consistent with those published by Albeshr and Alrefaei (2020) [8], who reported that infected samples of *T*. *gallinae* sequence C isolated from pigeons and chickens exhibited apparent gross lesions or evident clinical signs. However, more extensive studies are required to determine the epidemiology and pathogenesis of *T*. *gallinae* infecting Saudi falcons, which might elucidate to what extent, if any, virulent traits can be ascribed to genetic lineages of this parasite.

*Trichomonas gallinae* commonly infects falcons in Saudi Arabia and the Middle East [11,30], and trichomonosis is often linked to the practice of feeding falcons live or freshly killed pigeons, in particular domestic pigeons *(Columba livia)* [11,12]. In Saudi Arabia, falconers typically buy such pigeons from a poultry market or catch them on farms. Albeshr and Alrefaei (2020) [8] recently reported that in Riyadh, more than 63% of feral pigeons for sale in Al-Azizia’s poultry market and 17% of wild pigeons caught on the south and south-western sides of nearby Wadi-Hanifa were infected by *T*. *gallinae*. Many studies have reported that populations of *Columba livia*, as well as other members of the order Columbiformes, are important reservoirs of the protozoan *T*. *gallinae* [5,9,19,31–33]. There is a need to extend screening to additional falcon species worldwide to evaluate the genetic diversity of trichomonas in both falcons and their prey and investigate related clinical and subclinical impacts and potential pathogenicity.

## Supporting information

S1 Fig Agarose Gel Electrophoresis of the PCR product of isolates of *T*. *gallinae*. (TIF)
