# Supplementary figures and images for: Molecular detection and genetic characterization of Trichomonas gallinae in falcons in Saudi Arabia
Source: PLoS One. 2020 Oct 29;15(10):e0241411. doi: 10.1371/journal.pone.0241411 (PMC7595344; doi:10.1371/journal.pone.0241411)

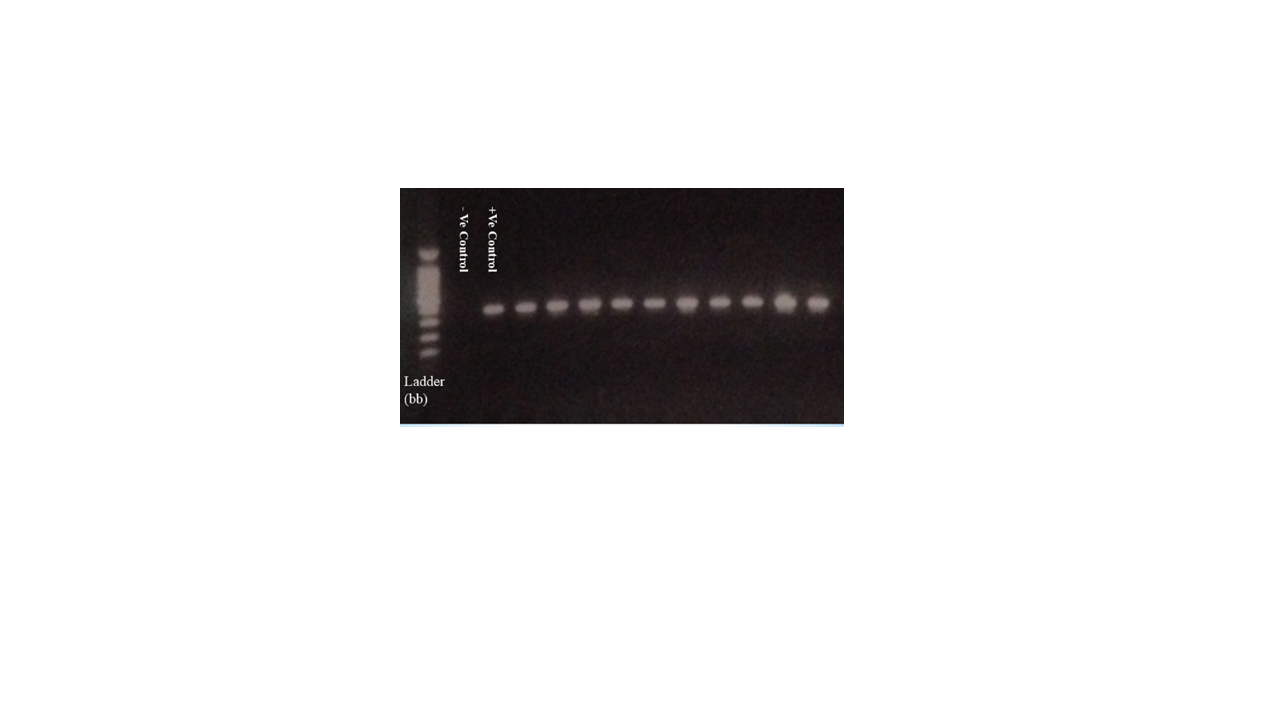

Supplement: S1 Fig — (TIF) [file pone.0241411.s001.tif]
